# A Novel Method for Obtaining Well-Separated Mn_3_O_4_ Nanocrystallites Deposited on the Surface of Spherical Silica

**DOI:** 10.3390/ijms26178413

**Published:** 2025-08-29

**Authors:** Oleksandr Pastukh, Magdalena Laskowska, Jarosław Jędryka, Maciej Zubko, Łukasz Laskowski

**Affiliations:** 1 Institute of Nuclear Physics Polish Academy of Sciences, PL-31342 Krakow, Poland; magdalena.laskowska@ifj.edu.pl; 2 Faculty of Electrical Engineering, Czestochowa University of Technology, Al. Armii Krajowej 17, 42-200 Czestochowa, Poland; jaroslaw.jedryka@pcz.pl; 3 Institute of Materials Engineering, University of Silesia in Katowice, 75 Pułku Piechoty 1A St., 41-500 Chorzów, Poland; maciej.zubko@us.edu.pl

**Keywords:** manganese oxide, nanocrystals, hausmannite, nonlinear optics

## Abstract

Manganese oxides have recently gained a lot of interest from scientists due to their unique structural, magnetic and optical properties, which make them favorable for diverse nanotechnological applications. Most applications, however, require stable and well-dispersed nanoparticles of nanometer size. Therefore, in this work, we show a procedure for obtaining separated crystallites of manganese oxide ${\mathrm{Mn}}_{3}$ $O_{4}$ on the surface of spherical silica carriers. The morphology and properties of nanoparticles were analyzed based on transmission electron microscopy observations, Raman spectroscopy, and low-temperature SQUID measurements. The analysis of results revealed the formation of well-dispersed ${\mathrm{Mn}}_{3}$$O_{4}$ nanoparticles with an average size of approximately 9 nm. The magnetic measurements confirmed the characteristic critical temperature, and a narrow hysteresis loop appeared due to the surface anisotropy of nanoparticles. It was additionally demonstrated that such small nanoparticles possess pronounced nonlinear optical properties, as evidenced by strong signals of second and third harmonic generation. The obtained results fully confirmed the synthesis assumptions and offer promising prospects for the development of a new class of highly optically active manganese-based nanocomposites.

## 1. Introduction

Manganese oxides are versatile materials, possessing many oxidation states and having a wide range of applications, such as high-density magnetic storage media, anode materials in lithium-ion batteries, catalysts, ion exchange, solar energy conversion, medicinal materials, and many others [1,2,3,4]. Among different manganese oxides, ${\mathrm{Mn}}_{3}$ $O_{4}$ (hausmannite) attracts particular interest because of its distinctive structural and electronic features with interesting magnetic and electrochemical properties [5,6]. Hausmannite nanoparticles show ferrimagnetic behavior with a Curie temperature value, which depends on the size of the nanoparticles [7]. Such compounds have been widely used as the main source of ferrite materials and ferrofluids [8], with extensive applications in electronic devices, sensors [9], and magnetic data storage [10]. ${\mathrm{Mn}}_{3}$$O_{4}$ nanoparticles are also interesting from the point of view of their catalytic properties. Such a material has been used as an active catalyst for the reduction of nitrobenzene or oxidation of methane [11,12]. Moreover, hausmannite nanoparticles are effective and inexpensive catalysts to limit the emission of NO*x* and CO [13,14], which provides a powerful method for controlling air pollution. Also, ${\mathrm{Mn}}_{3}$$O_{4}$ nanoparticles display great biological properties and low toxicity [15,16] and therefore can be used in pharmaceuticals.

The important point of the practical applications of manganese oxide is that most of them require the use of uniform and well-dispersed chemically stable nanometer-scale particles [17]. Obtaining such structures with controlled sizes in the nanoscale (in a pure form, without aggregations, with uniform size and shape) opens up an absolutely new applicative potential of the material, unreachable for bulk crystals. The attempts for the synthesis of separated nanosized ${\mathrm{Mn}}_{3}$$O_{4}$ nanoparticles described in the literature were based in most cases on using thermal hydrolysis or the organic phase decomposition of manganese salts [7,18,19]. Many other synthesis methods were also applied, including, for instance, dissolution [20] or autoxidation [21] of manganese acetate in room temperature, chemical precipitation [22], the sol–gel method [23], microwave or ultrasonic irradiation [24,25], among others. On the other hand, more promising and successful methods for the stabilization and obtaining of higher monodispersity of manganese oxide nanoparticles were based on their deposition and regular organization on surfaces, or incorporation into polymer, ceramic, glass, or zeolite matrices [26,27]. It has been reported that the formation of the nanoparticles of hausmannite is possible through methods such as synthesis inside mesoporous channels of silica matrices [14,28] and hollow carbon spheres [29], anchoring onto graphene sheets [30], or applying the incorporation into carbonaceous materials such as graphene and carbon nanotubes [31,32].

In this work, we present an alternative method for obtaining ultramicroscopic hausmannite nanoparticles on the surface of silica, which are characterized by high size uniformity and relatively regular distribution (nanocrystals are well separated). The main idea of the current study is based on the use of ${\mathrm{Mn}}_{12}$-stearate (${\mathrm{Mn}}_{12}$-st) single-molecule magnets anchored to the surface of spherical silica nanoparticles as a precursor for the formation of manganese oxide nanoparticles by the thermal treatment process. It has been previously shown that such molecules can be separated and regularly distributed on the spherical silica surface [33]. Such a factor is key for the formation of separated and well-dispersed manganese oxide nanoparticles.

However, the structure of the obtained material poses a significant problem in terms of its accurate examination. Due to the small size of ${\mathrm{Mn}}_{3}$$O_{4}$ nanocrystals, confirming their structure is a considerable challenge. Therefore, we present an experimental procedure for examining the obtained material.

## 2. Results and Discussion

**Figure 1:**
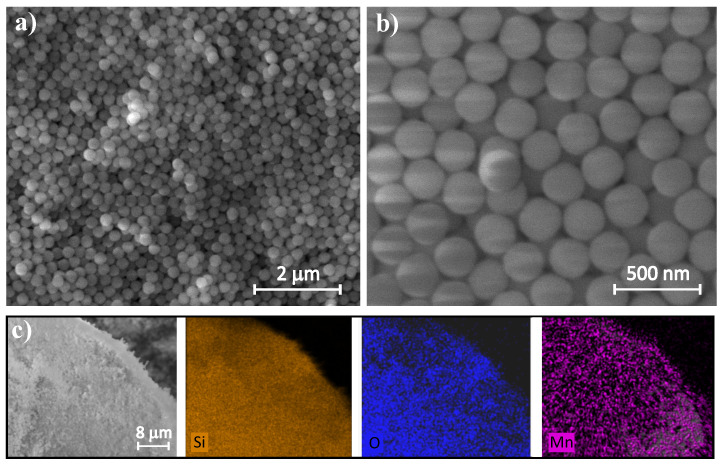
SEM images of spherical silica nanoparticles at different magnification (**a**,**b**) and EDS elemental maps showing Si, O and Mn distribution (**c**).

Figure 1a,b represent SEM images of a substrate material of manganese oxide, namely spherical silica nanoparticles. The silica NPs appear uniform in size and are monodispersed, indicating a well-controlled synthesis process. The average diameter of silica spheres is 300 nm, as assumed during synthesis. The analysis of EDS elemental mapping (Figure 1c) reveals a homogeneous distribution of both manganese (Mn) and oxygen (O) across the sample. No visible agglomerates are observed, confirming the even dispersion of manganese oxide nanoparticles on the silica support. An EDS spectrum and quantitative analysis of the atomic and weight concentration of Si and Mn show a small fraction of manganese and a dominant contribution of silica substrate in the sample (see Figure S1 and Table S1 of Supplementary Information).

**Figure 2:**
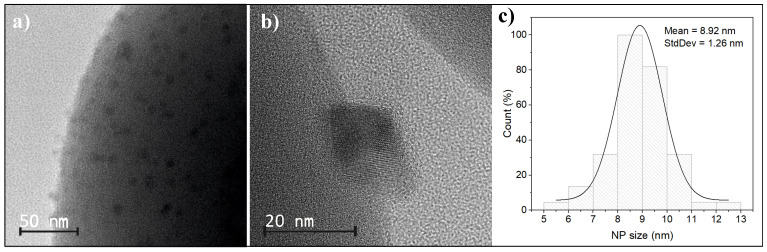
The TEM images of spherical silica material with deposited manganese oxide NPs at low-magnification (**a**), showing the surface of the silica sphere, and high magnification (**b**), showing individual manganese oxide nanoparticle. Figure (**c**) shows the size distribution of manganese oxide particles.

The transmission electron microscopy of functionalized spherical silica nanoparticles after the calcination process is shown in Figure 2a–c. It should be noted that obtaining TEM images to visualize both Mn_12_ anchored on the silica surface and nanocrystals on silica spheres is very difficult, as it requires a slightly out-of-focus observation of the materials, which is a technique that has been developed through our extensive experience in observing this type of material. Therefore, it is not possible to obtain images of ideal quality. The use of scanning transmission electron microscopy (STEM) is also not possible because the focusing of the electron beam destroys the organic structures present in Mn_12_, which are responsible for their anchoring on the silica surface, and even causes the structure of the obtained crystallites to ‘flow’. Therefore, in this work, we present TEM images that, despite slight defocusing, excellently illustrate the obtained materials. As can be seen, the manganese oxide nanoparticles are well separated and evenly distributed on the surface after thermal treatment. This was achieved by the controlled anchoring of ${\mathrm{Mn}}_{12}$-st clusters on the surface by propyl-carbon units, which caused the dispersion of molecular magnet binding sites [34]. As a result, the formed manganese oxide nanoparticles do not aggregate but remain well separated on the silica surface. The obtained individual nanoparticles possess a mean size of about 9 nm with a size deviation that does not exceed 1.2 nm.

**Figure 3:**
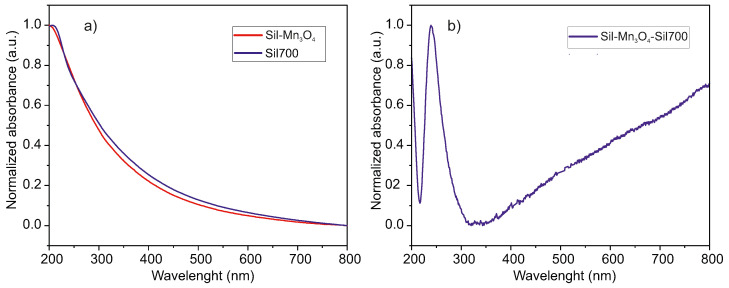
The UV-Vis absorption spectra of spherical silica particles decorated with ${\mathrm{Mn}}_{3}$$O_{4}$ NPs and pure spherical silica particles calcined at 700 °C (**a**) together with the graph obtained by subtracting the substrate spectrum from the spectrum of the tested nanocomposite (**b**).

Silica-containing manganese oxide nanoparticles were examined using UV–Vis analysis. Figure 3a shows the UV–Vis spectrum of pure silica substrates after thermal treatment (labeled Sil700) and functionalized silica after thermal treatment (labeled ${Sil-Mn}_{3}$$O_{4}$). Additionally, a graph obtained by subtracting the substrate spectrum from the spectrum of the tested nanocomposite is shown (Figure 3b). Looking at the latter, it is clear that the absorption maximum occurs at values typical for ${\mathrm{Mn}}_{3}$$O_{4}$ nanoparticles reported in the literature [15,35,36,37], i.e., at around 240 nm. UV–Vis analysis confirmed the presence of ${\mathrm{Mn}}_{3}$$O_{4}$ in the sample and ruled out the presence of other manganese oxide phases, as no additional absorption bands characteristic of alternative oxide forms were detected in the spectrum.

**Figure 4:**
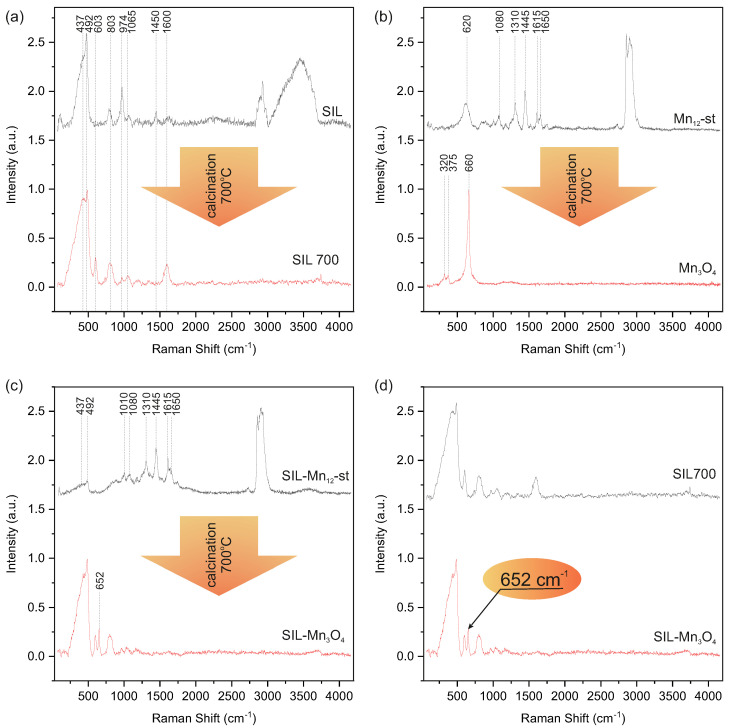
Raman spectra of spherical silica particles before (SIL) and after calcination (SIL700) (**a**), ${\mathrm{Mn}}_{1}2$-st before and after calcination (${\mathrm{Mn}}_{3}$$O_{4}$) (**b**), spherical silica particles decorated with ${\mathrm{Mn}}_{1}2$-st before (${\mathrm{SIL-Mn}}_{1}2$-st) and after calcination (${\mathrm{SIL-Mn}}_{3}$$O_{4}$) (**c**) and The comparison of SIL700 and ${\mathrm{SIL-Mn}}_{3}$$O_{4}$ spectra (**d**).

In order to perform a reliable Raman spectroscopy analysis of the obtained silica particles decorated with ${\mathrm{Mn}}_{3}$$O_{4}$ nanocrystals, a series of reference samples obtained at subsequent stages of the preparation procedure were also investigated. Since the final material was obtained by calcination of spherical silica systems with ${\mathrm{Mn}}_{12}$-st molecules deposited on their surface, we analyzed changes in the Raman spectrum of individual components of this system as a result of calcination. Figure 4a shows the spectra of spherical silica particles before and after calcination in air at 700 °C. Analysis of these spectra indicates significant changes in the amorphous structure of silica during calcination. A spectrum of spherical silica particles prior to calcination (SIL) contains bands typical of amorphous silica. The overlapping bands at around 430 and 492 ${\mathrm{cm}}^{-1}$ are attributed to six- and four-membered rings, respectively [38]. The band at 803 ${\mathrm{cm}}^{-1}$ is associated with the symmetric stretching and bending modes of the ${\mathrm{SiO}}_{4}$ units, while the less intense band at 1065 ${\mathrm{cm}}^{-1}$ originates from the anti-symmetric stretching vibration of the tetrahedral ${SiO}_{4}$ units [39]. At 974 ${\mathrm{cm}}^{-1}$, there is a band related to silica tetrahedra with two non-bridging oxygens with silicon–oxygen stretching modes of Si-OH groups [40,41]. Residues of ammonia bound inside silica during synthesis are visible in the form of a band at 1450 ${\mathrm{cm}}^{-1}$ originating from ${NH}_{4}^{+}$ ions [42]. The other bands observed in this spectrum at 1600 ${\mathrm{cm}}^{-1}$ and in the region 2800–3700 ${\mathrm{cm}}^{-1}$ should be attributed to water molecules bounded in the silica structure and to silanol groups on its surface [43]. A comparison of this spectrum with that of calcined silica spheres (SIL700) shows that calcination caused significant changes in the silica structure as evidenced by the new band at 605 ${\mathrm{cm}}^{-1}$. This feature originates from the cyclic trisiloxane rings as a result of the condensation of surface silanols during the thermal treatment [44]. The rest of the SIL700 spectrum resembles the spectrum of SIL, but the bands at 437, 803, 974, and 1603 ${\mathrm{cm}}^{-1}$ differ in their intensities. While the intensity of the bands at 437, 803, and 1603 ${\mathrm{cm}}^{-1}$ increases after calcination as a result of the polycondensation process, which creates additional Si-O-Si bonds in the structure as a result of thermal treatment, the band at 974 ${\mathrm{cm}}^{-1}$ decreases in intensity due to the reduction in silanol groups during calcination [41,45].

Figure 4b shows a comparison of the spectra of bulk ${\mathrm{Mn}}_{12}$-st before and after calcination. Presented comparison clearly shows that starting with the bulk form of ${\mathrm{Mn}}_{12}$-st, through thermal treatment in air at a temperature of 700 °C, we obtain pure ${\mathrm{Mn}}_{3}$$O_{4}$. The spectrum of ${\mathrm{Mn}}_{12}$-st before heat treatment shows distinct bands originating from organic stearin ligands and Mn-O bonds in the molecule core. The broad maximum at 620 ${\mathrm{cm}}^{-1}$ is a result of Mn-O vibrations. The band at 1080 ${\mathrm{cm}}^{-1}$ finds its origin in the C-C stretching modes [46], while bands at ∼1310 ${\mathrm{cm}}^{-1}$ and ∼1445 ${\mathrm{cm}}^{-1}$ derive from the twisting of numerous ${\mathrm{CH}}_{2}$ groups in the stearic acid chains [46]. The last two bands in this region, at 1615 and 1650 ${\mathrm{cm}}^{-1}$, originate from the asymmetric C-O stretching modes and correlate with the formation of the ${\mathrm{Mn}}_{12}$-st complex with the ligated carboxylates in bidentate stearic acid [47] and from bending H-O-H vibrations in water molecules [48], respectively. The last region from 2800 to 3000 ${\mathrm{cm}}^{-1}$ contains the most intense bands, indicating the stretching modes of the methylene and methyl species present in the stearate ligands [49]. Per contra, the spectrum of the sample after calcination contains only three clearly distinguished Raman bands at 320, 375, and 660 ${\mathrm{cm}}^{-1}$, which are similar to those reported for hausmannite ${\mathrm{Mn}}_{3}$$O_{4}$ [50,51]. Moreover, considering the features of maxima observed in this spectrum, such as sharp and symmetric peaks with positions typical for the hausmannite spectrum, it can be concluded that calcination of the bulk form of ${\mathrm{Mn}}_{12}$-st leads to larger forms of ${\mathrm{Mn}}_{3}$$O_{4}$ than nanoparticles [52].

Figure 4c shows a comparison of the spectra of silica with ${\mathrm{Mn}}_{12}$-st molecules deposited on its surface (${\mathrm{SIL-Mn}}_{12}$-st) and the same material after calcination (${\mathrm{SIL-Mn}}_{3}$$O_{4}$), which, as shown by the analysis of both spectra, is a silica decorated with ${\mathrm{Mn}}_{3}$$O_{4}$ nanocrystals. The upper (black) spectrum in Figure 4c exhibits all features typical for ${\mathrm{Mn}}_{12}$-st as well as silica. The first part of this spectrum is dominated by bands originating from silica, which overlap the bands resulting from Mn-O vibrations, while the region 1000–2000 ${\mathrm{cm}}^{-1}$ presents bands originating form stearate ligands. Moreover, in the region from 2800 to 3000 ${\mathrm{cm}}^{-1}$, bands indicating the stretching modes of the methylene and methyl species present in the stearate ligands can be observed, as in bukl form of ${\mathrm{Mn}}_{12}$-st [49]. However, the spectrum of the sample after calcination changes significantly. The lower spectrum (red) in Figure 4c contains only bands characteristic of calcined silica (lower spectrum in Figure 4a) and one additional band at around 652 ${\mathrm{cm}}^{-1}$. This band can be attributed to vibrations of the hausmannite structure, which confirms the transformation of ${\mathrm{Mn}}_{12}$-st molecules into ${\mathrm{Mn}}_{3}$$O_{4}$ nanocrystals. The position of the band assigned to the ${\mathrm{Mn}}_{3}$$O_{4}$ structure at 652 ${\mathrm{cm}}^{-1}$ in this spectrum can also be a source of information about the size of the nanocrystals. According to reports in the literature, such a red shift is associated with a reduction in crystallite dimensions. Referring to the research of J. Zuo et al., we can assume that the nanocrystallites deposited on silica are smaller than the nanocrystallites obtained from bulk ${\mathrm{Mn}}_{12}$-st and are no larger than 40 nm [52]. The stacked Raman spectra showing a comparison of peak location and intensities of ${\mathrm{SIL-Mn}}_{3}$$O_{4}$ and ${\mathrm{SIL-Mn}}_{12}$ with the reference samples is presented in Figure S2 of the Supplementary Information. Figure 4d summarizes the analysis in a comparison of the spectra of pure silica after calcination (SIL700) and silica decorated with ${\mathrm{Mn}}_{3}$$O_{4}$ nanocrystals (${\mathrm{SIL-Mn}}_{3}$$O_{4}$). This comparison shows that both samples differ in the presence of a distinct band at 652 ${\mathrm{cm}}^{-1}$ originating from the ${\mathrm{Mn}}_{3}$$O_{4}$ structure. In summary, Raman spectroscopy confirmed the formation of ${\mathrm{Mn}}_{3}$$O_{4}$ nanocrystals on the surface of spherical silica particles during calcination. Furthermore, the method of separating ${\mathrm{Mn}}_{12}$-st molecules on the surface of silica leads to the formation of very small nanocrystals with sizes below 40 nm, which is impossible to achieve by direct calcination of ${\mathrm{Mn}}_{12}$-st in bulk form.

**Figure 5:**
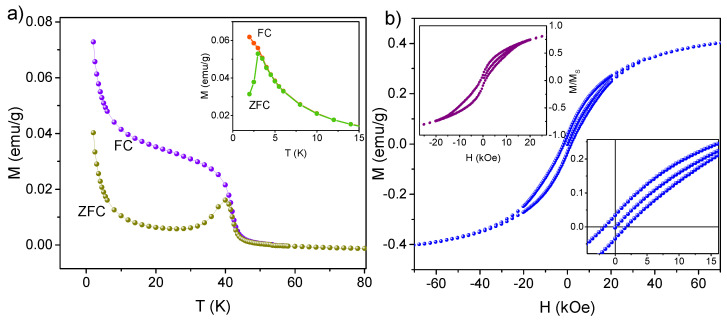
ZFC and FC magnetic susceptibility as a function of temperature at the applied field of $H_{DC}$ = 2 kOe (**a**) and magnetic hysteresis loop measured at T = 2.0 K (**b**) for the spherical silica containing manganese oxide nanoparticles. The insets of (**a**,**b**) show temperature-dependent and field-dependent magnetization for the sample prior to calcination (i.e., ${\mathrm{SIL-Mn}}_{12}$-st), respectively.

As can be seen, the structural characterization of the synthesized manganese oxide nanoparticles presents significant challenges due to their extremely small volume. Therefore, additional information on their properties was obtained based on magnetic measurements, carried out for a sample ${\mathrm{SIL-Mn}}_{3}$$O_{4}$, as well as the sample before calcination, as a reference. First, the temperature-dependent magnetization measurements were performed by applying a constant DC external field with an amplitude of H = 2 kOe. In this procedure, the sample was initially cooled to 2.0 K without an external field and then, with the field applied, heated to 300 K (ZFC curve in Figure 5a). Following this, the sample was cooled again in the presence of a magnetic field, and a change in net sample magnetization was recorded (FC curve in Figure 5a). The ZFC/FC measurements of ${\mathrm{SIL-Mn}}_{12}$-st sample (see inset of Figure 5a) indicate the presence of blocking temperature $T_{B}$ = 3.0 K, which is typical for ${\mathrm{Mn}}_{12}$-st SMMs [47]. In contrast, for the sample after heat treatment, both ZFC and FC curves exhibit near-zero magnetization values at room temperature, followed by a pronounced increase around 40 K, suggesting a transition from paramagnetic to ferromagnetic behavior. Notably, the ZFC curve shows a distinct peak at T = 40 K, indicating the existence of a critical temperature. This value aligns well with previously reported critical temperature for ${\mathrm{Mn}}_{3}$$O_{4}$ nanoparticles, which are slightly lower than that of the bulk material ($T_{c}$ = 43 K), and is consistent with findings for ${\mathrm{Mn}}_{3}$$O_{4}$ nanoparticles with sizes of 8–10 nm [7,28,53]. Let us note that the temperature at which the maximum of the ZFC curve appears and the temperature where ZFC and FC curves superimpose are very close to each other (40 K and 42 K, respectively). This indicates a very narrow ${\mathrm{Mn}}_{3}$$O_{4}$ particle size distribution in the sample [54] which is in agreement with the TEM observations. As we can also see, neither the ZFC nor the FC curves exhibit saturation at lower temperatures, and start to rise above approximately 10 K. Such behavior was previously observed in ${\mathrm{Mn}}_{3}$$O_{4}$ nanoparticles dispersed in a polymer [55] or distributed inside the mesoporous silica matrix [28] and is typically attributed to the absence of significant magnetic interactions between individual nanoparticles [56].

Additionally, the dependence of magnetization on the applied field was measured at low temperature (T = 2.0 K). The isothermal magnetization of the sample before calcination shows the presence of a relatively wide hysteresis loop with a butterfly shape, which is typical for surface-deposited ${\mathrm{Mn}}_{12}$ derivatives [57]. On the one hand, following Figure 5b, the sample ${\mathrm{SIL-Mn}}_{3}$$O_{4}$ exhibits a narrow hysteresis loop with a coercive field of about 1.6 kOe. The obtained value is nearly four times lower than that reported for 13 nm ${\mathrm{Mn}}_{3}$$O_{4}$ aggregated nanoparticles ($H_{c}$ = 7 kOe for T = 3.0 K [58]), confirming that there are no interactions between particles. On the other hand, the observed hysteresis can be attributed to the pronounced shape and surface anisotropy of nanoparticles [56], which arise due to their confined size. Analysis of the high-field magnetization reveals that the sample does not exhibit saturation even at an applied magnetic field of 7 T. This appears as a result of the ${\mathrm{Mn}}_{3}$$O_{4}$ nanoparticle distribution on the surface of spherical silica, which hinders complete alignment of individual spins in the presence of an external magnetic field.

**Figure 6:**
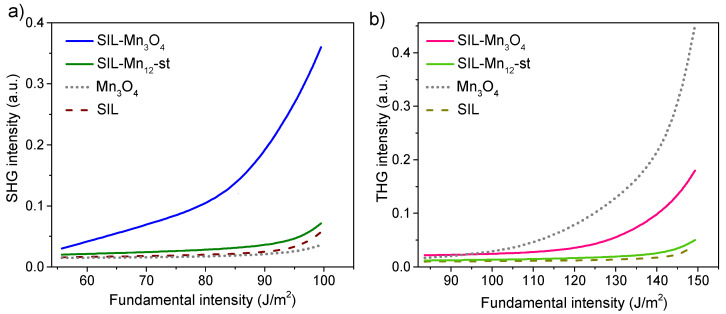
Dependence of the SHG (**a**) and THG (**b**) signal on the fundamental laser intensity for the silica containing manganese oxide nanoparticles and reference samples (pure silica, bulk ${\mathrm{Mn}}_{3}$$O_{4}$, and silica with deposited molecules ${\mathrm{Mn}}_{12}$-st).

Different studies have demonstrated the potential of using nanostructured silica as a substrate for the deposition and uniform distribution of optically active functional units [59,60]. The enhanced optical nonlinearities observed in such systems can be attributed to the spatial confinement effect of optical units presented in nanocomposites and their nanostructured form, in contrast to their bulk counterparts [61]. Therefore, the nonlinear–optical properties of the studied material were also analyzed. The dependence of second harmonic generation and third harmonic generation on the fundamental intensity is shown in Figure 6. The SHG and THG signals from manganese oxide nanoparticles deposited on a silica surface were compared with reference samples: pure spherical silica (SIL), functionalized silica before calcination (${\mathrm{SIL-Mn}}_{12}$-st), and bulk ${\mathrm{Mn}}_{3}$$O_{4}$.

As can be seen, the pure spherical silica sample does not exhibit any significant SHG or THG signals. The functionalized sample prior to calcination shows a slightly enhanced SHG and THG response; however, the signal remains relatively weak and is only detectable at higher excitation intensities. In contrast, the thermally treated (calcined) sample exhibits a significantly higher nonlinear optical response. For instance, in the case of SHG (Figure 6a), the signal increases exponentially with rising fundamental intensity (FI), reaching a value 12 times higher at a maximum intensity of 100 J/$m^{2}$ compared to the initial value at 55 J/$m^{2}$. A similar trend is observed for THG (Figure 6b), although the increase is approximately sevenfold (at FI = 150 J/$m^{2}$) relative to the initial intensity of 110 J/$m^{2}$.

It is worth noting that a similar amplitude of SHG and THG responses as a function of fundamental laser intensity has been observed for copper phosphate nanostructures and nickel pyrophosphate nanocrystals embedded within SBA-15 mesoporous silica matrices [62,63]. These structures, however, were shown to possess strong electronic charge density distributions, which contribute to their enhanced optical nonlinearities. In contrast, for the studied materials, since the observed nanoparticles are about ten nanometers in size, the enhancement of the nonlinear optical response can be mainly attributed to the surface effects. As reported in the literature, the surface of nanoparticles plays a critical role in defining their second- and third-order nonlinear optical properties [64,65]. In this context, the increase in hyperpolarizability per unit volume is largely due to surface contributions, which arise from the breaking of centrosymmetry at the nanoparticle surface and deviations from centrosymmetric geometry. At the nanoparticle surface, the atomic environment is inherently asymmetric, leading to polarization of the chemical bonds between atoms [66]. Furthermore, the local electric field near the surface can exhibit significant spatial variation, further enhancing hyperpolarization effects. This variation may therefore contribute to an overall increase in the nonlinear optical response of the material [67].

In this context, it is important to analyze the NLO response of bulk ${\mathrm{Mn}}_{3}$$O_{4}$. As can be seen in Figure 6, the second-harmonic component is practically negligible over the entire range of fundamental power densities. This result is consistent with expectations, since the hausmannite structure is centrosymmetric (space group $I4_{1}/amd$), and therefore, the electric-dipole bulk second-order (third-rank) susceptibility tensor $\chi_{ijk}^{\left(2\right)}$ reaches zero [68,69]. In centrosymmetric media, residual SHG can only arise from magnetic-dipole or electric-quadrupole contributions, or from surfaces and interfaces where inversion symmetry is broken. This supports our conclusion that the SHG signal observed in the nanocomposite originates from surface-related effects at the nanoscale, where local symmetry breaking, strain, and defects can induce a measurable $\chi^{\left(2\right)}$ response even if the bulk material is centrosymmetric [67].

The situation is different for the third-harmonic component. For bulk hausmannite, it is clearly detectable, as expected (Figure 6), since third-order nonlinear processes described by the fourth-rank tensor $\chi_{ijkl}^{\left(3\right)}$ do not require acentric crystal symmetry. While comprehensive experimental data for bulk single-crystal ${\mathrm{Mn}}_{3}$$O_{4}$ are scarce, estimations using semi-empirical models such as the Ticha–Tichy approach (combined with Miller’s rule) for thin films suggest that $\chi^{\left(3\right)}$ may be on the order of $10^{-13}$ esu, typical for transition metal oxides [70]. It should be noted, however, that the amount of pure ${\mathrm{Mn}}_{3}$$O_{4}$ in our composite samples is significantly lower than in the bulk reference material. Therefore, the observed THG signal in the nanocomposite represents a substantial enhancement when considered relative to the effective volume of the active material.

## 3. Materials and Methods

### 3.1. Synthesis Procedure

**Figure 7:**
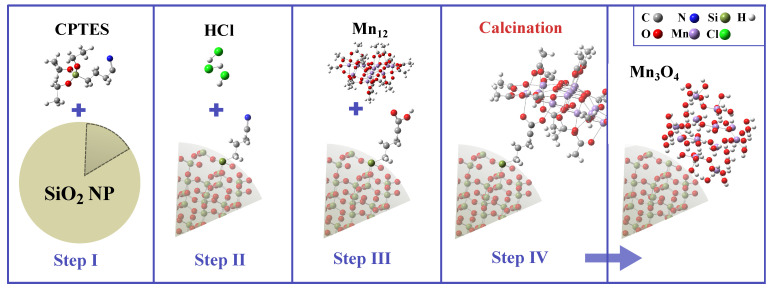
Synthesis procedure for the obtaining of manganese oxide nanoparticles on the surface of spherical silica. Assumed steps: grafting (I), hydrolysis (II), functionalization (III), and calcination (IV).

The schematic representation of the synthesis procedure is presented in Figure 7. The substrate material, consisting of spherical silica nanoparticles with an assumed diameter of 300 nm, was initially synthesized using the Stöber protocol [71,72]. In the first step (Step I) of the functionalization process, the silica spheres were grafted with cyanopropyl groups. To achieve this, a solution of 3-cyanopropyltriethoxysilane (CPTES) in dichloromethane (2% *v*/*v*) was prepared. Dry silica nanoparticles were then added to this solution, and the resulting suspension was stirred vigorously under reflux for 24 h under an argon atmosphere. The pre-functionalized silica was recovered by centrifugation and thoroughly washed with dichloromethane.

In the second step (Step II), the nitrile groups of the grafted cyanopropyl moieties were hydrolyzed to carboxylic acid groups using a 6 M hydrochloric acid solution in a 1:1 (*v*/*v*) mixture of water and acetone. The reaction was carried out under reflux overnight. The resulting product was obtained by centrifugation and washed with acetone until the pH was neutral.

In the third step (Step III), silica spheres containing carbonic acid anchoring units at the surface were functionalized with ${\mathrm{Mn}}_{12}$-st molecules. The synthesis of ${\mathrm{Mn}}_{12}$-st was performed according to the protocol described elsewhere [47,73]. The functionalization was conducted by mixing the reagents in dichloromethane and stirring the solution overnight at room temperature under an argon atmosphere. The resulting powder was separated by centrifugation, washed with dichloromethane, and dried under vacuum.

In the final step (Step IV), calcination of the ${\mathrm{Mn}}_{12}$-functionalized silica spheres was performed at 973 K (700 °C) under air for 7 h (heating rate of 2 °C $\min^{-1}$). This thermal treatment led to the formation of manganese oxide nanoparticles anchored on the surface of the spherical silica particles.

### 3.2. Characterization Methods

The scanning electron microscope (SEM) Tescan Vega 3 (Brno, Czech Republic) was used to record micrographs. All tested samples, in powdered form, were mounted onto a specialized SEM sample stub, covered with conductive carbon tape to minimize charging effects during imaging. To achieve optimal dispersion and avoid agglomeration, the powder was gently pressed into the tape using a clean, non-metallic tool, ensuring stable positioning of the particles under the electron beam. Samples were imaged in high-vacuum mode at an accelerating voltage of 15 kV using a secondary electron (SE) detector. The Bruker QUANTAX energy-dispersive spectrometer (Berlin, Germany) (EDS) was used to acquire elemental maps using a high count rate and 15 keV beam energy.

The transmission electron microscope (TEM) FEI Tecnai G2 20 X-TWIN (Hillsboro, OR, USA), equipped with the emission source LaB6 and a CCD camera (FEI Eagle 2K), was used to record micrographs with high magnification. The average diameter of the silica spheres and the size distribution of manganese oxide nanoparticles were measured from micrographs using ImageJ (v. 1.54m, National Institutes of Health, Bethesda (MD), USA) software [74]. The size measurements were performed along the major dimension of the nanoparticle on a sufficient number of NPs to obtain a statistically relevant distribution.

Raman spectra were collected at 22 °C using Raman microscope WITec alpha 300R (Ulm, Germany) equipped with a High-Performance Low-dark current CCD Camera ANDOR iVac, a spectrometer UHTS300 SMFC VIS-NIR with focal length 300 mm, and grating 600 g/mm BLZ = 500 nm. A Zeiss EC Epiplan-Neofluar Dic 100×/0.9 objective (Oberkochen, Germany) was used to focus the incident laser light onto the sample. All spectra were accumulated by 50 scans with an integration time of 5 s using laser power from 2 to 20 mW for 532 nm laser. The samples after calcination were investigated using 20 mW laser power while ${\mathrm{Mn}}_{12}$-st and ${\mathrm{SIL-Mn}}_{12}$-st samples were investigated using 2 mW laser power.

UV-Vis absorption spectra were recorded on Shimadzu UV-2600 UV-Vis spectrophotometer (Kyoto, Japan) over 200–800 nm with a 1 nm data interval. Aqueous suspensions (1 mg· ${\mathrm{mL}}^{-1}$) were prepared in ultrapure water, and spectra were collected using 1 cm quartz cuvettes. Baseline correction was performed using an ultrapure water blank. Measurements were carried out at room temperature.

Magnetic properties were studied with the superconducting quantum interference device (SQUID) magnetometer Quantum Design MPMS (San Diego, CA, USA). Isothermal magnetization M(H) was assessed at a temperature of 2.0 K, across a range of −70 kOe to 70 kOe of external magnetic field. DC magnetic susceptibility was recorded during zero-field cooling (ZFC) and field cooling (FC) measurements, over a temperature change of 2.0–100 K under an external magnetic field strength (H) of 2 kOe. The diamagnetic contribution from silica particles was deducted from the magnetic data.

Nonlinear optical properties of the samples, which include second and third harmonic generation signals (SHG and THG, respectively), were measured using Nd:YAG nanosecond laser generating light (Lannion, France) at 1064 nm with a pulse duration of about 10 ns and a pulse repetition rate of 10 Hz. Output signals were recorded using interference filters with 5 nm spectral width signals at 532 nm and 355 nm, respectively. NLO results were given for the optimized angle between the propagation of the primary beam and the sample surface. Recording was performed using a photomultiplier with a relaxation time of 1 ns, connected to a Tectronics oscilloscope (Tektronix, Beaverton, OR, USA) with a resolution of 1 GHz. To focus the primary beam more precisely on the sample, a 2 mm diaphragm was placed between the mirror and the sample. The nonlinear optical properties of the samples were studied based on the analysis of the dependence of SHG and THG on basic laser intensity.

## 4. Conclusions

In this study, we present a method for synthesizing small, well-dispersed crystallites of manganese oxide (${\mathrm{Mn}}_{3}$$O_{4}$) on spherical silica carriers. The performed structural- and magnetic-property studies confirm the formation of the desired nanocomposite material after the calcination of the ${\mathrm{Mn}}_{12}$-st magnetic molecules on the silica substrate. Based on the microscopic observations, the highly separated and well-dispersed nanoparticles of approximately 9 nm in size were observed on the silica surface. Raman spectroscopy studies revealed the structural and chemical composition of the obtained nanoparticles, specifically the formation of the ${\mathrm{Mn}}_{3}$$O_{4}$ phase after the calcination of initially deposited ${\mathrm{Mn}}_{12}$-st molecules. The magnetic measurements confirmed the characteristic critical temperature of manganese oxide nanoparticles (T = 40 K) based on the temperature-dependent measurements. Additionally, the dependence of magnetization on the applied field revealed a weak interparticle interaction and a significant influence of surface anisotropy on their magnetic behavior. Importantly, it was also demonstrated that the obtained manganese oxide nanoparticles on the silica surface show a significant nonlinear optical response. The key factor in increasing the second and third harmonic generation is related to the surface effects that appear due to the small size of nanoparticles. In such a way, obtained nanostructures show great potential for a range of technological applications, particularly in the fields of nanoelectronics, nanophononics, and nanomagnetism.

## Data Availability

All data presented are included within the article.

## Supplementary Materials

The following supporting information can be downloaded at https://www.mdpi.com/article/10.3390/ijms26178413/s1. Reference [72] is cited in Supplementary Materials.
