# Supplementary material for: A Novel Method for Obtaining Well-Separated Mn3O4 Nanocrystallites Deposited on the Surface of Spherical Silica
Source: Int J Mol Sci. 2025 Aug 29;26(17):8413. doi: 10.3390/ijms26178413 (PMC12428405; doi:10.3390/ijms26178413)
Supplement: Supplementary file 1 [file ijms-26-08413-s001.zip › ijms-3805026-SM.pdf]

A Novel Method for Obtaining Well-Separated Mn_3O_4 Nanocrystallites Deposited on the Surface of Spherical Silica

Oleksandr Pastukh [1], Magdalena Laskowska [1], Jarosław Jędryka [2], Maciej Zubko [3] and Lukasz Laskowski [1,*]

[1] Institute of Nuclear Physics Polish Academy of Sciences, PL-31342 Krakow, Poland

[2] Faculty of Electrical Engineering, Czestochowa University of Technology, Al. Armii Krajowej 17, 42-200 Czestochowa, Poland

[3] Institute of Materials Engineering, University of Silesia in Katowice, 75 Pułku Piechoty 1A St., 41-500 Chorzów, Poland

S1. EDS spectra details

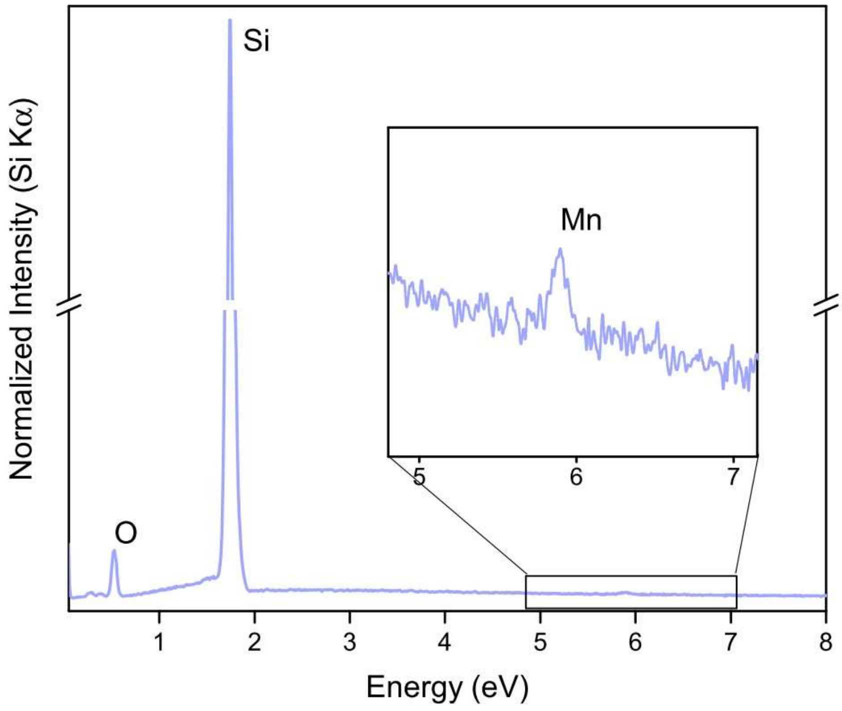

Figure S1. EDS spectra of spherical silica with deposited Mn_3O_4 nanoparticles.

Table S1. The manganese content in sample SIL-Mn_3O_4 obtained based on EDS analysis.

Element	Weight %	Atomic %
Si	99.13	99.55
Mn	0.87	0.45

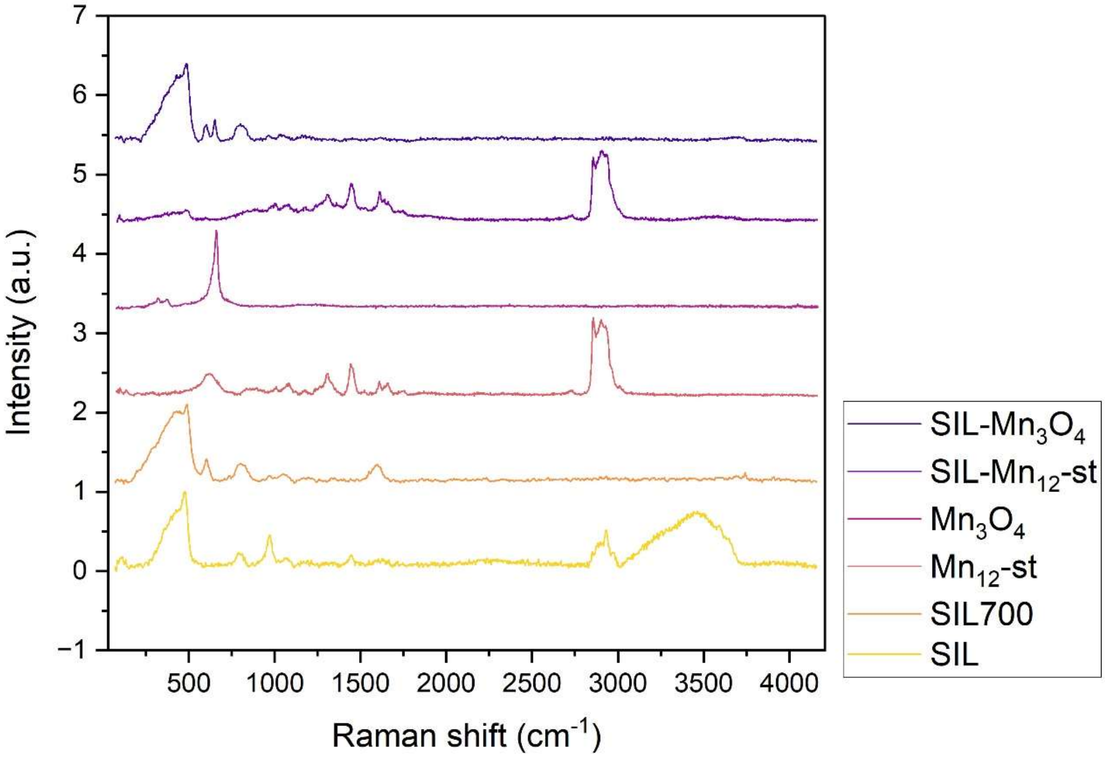

Figure S2. Stacked Raman spectra of silica particles with Mn_3O_4 NPs, silica particles with Mn_{12}-st molecules and reference samples.

S3. X-Ray Diffraction

X-ray powder diffraction (XRD) measurements were performed to evaluate the crystallinity and crystal structure of the obtained materials.

The experiment was conducted using Bruker D8 Advance diffractometer with CuKα radiation (λ = 1.5418 Å) and LynxEye detector, operating at 40 kV and 40 mA. The XRD studies of silica were performed in conventional Bragg-Brentano configuration for the range of 2θ angles from 10° to 85° with a size step of 0.01° and a time step of 5 s. The studied powder samples were compacted within the holders to form disks. Each specimen was subjected to rotation during the measurements to collect data from the entire sample.

As shown in Figure S3, both the target nanocomposite containing Mn_3O_4 nanocrystals (SIL-Mn_3O_4) and the reference sample in the form of pure spherical silica (Sil) exhibit very similar XRD spectra. In both cases, only features typical of silica are visible, in the form of a broad peak around 22 degrees and the absence of any Bragg peaks [1]. In the case of pure silica, this result is not surprising, but in the case of a material containing nanocrystals, the result is surprising because the material contains small crystalline

structures, as revealed by transmission electron microscopy. However, in this case, two important facts must be taken into account. Firstly, the small size of the obtained crystallites (approximately 10 nm) and the ratio of the volume of crystallites to the volume of the substrate. There are a few crystallites in relation to the silica substrate. Both of these facts mean that the Bragg peaks originating from Mn_3O_4 crystallites are probably so small that they are hidden in the noise. For this reason, the XRD technique does not seem suitable for studying this type of system.

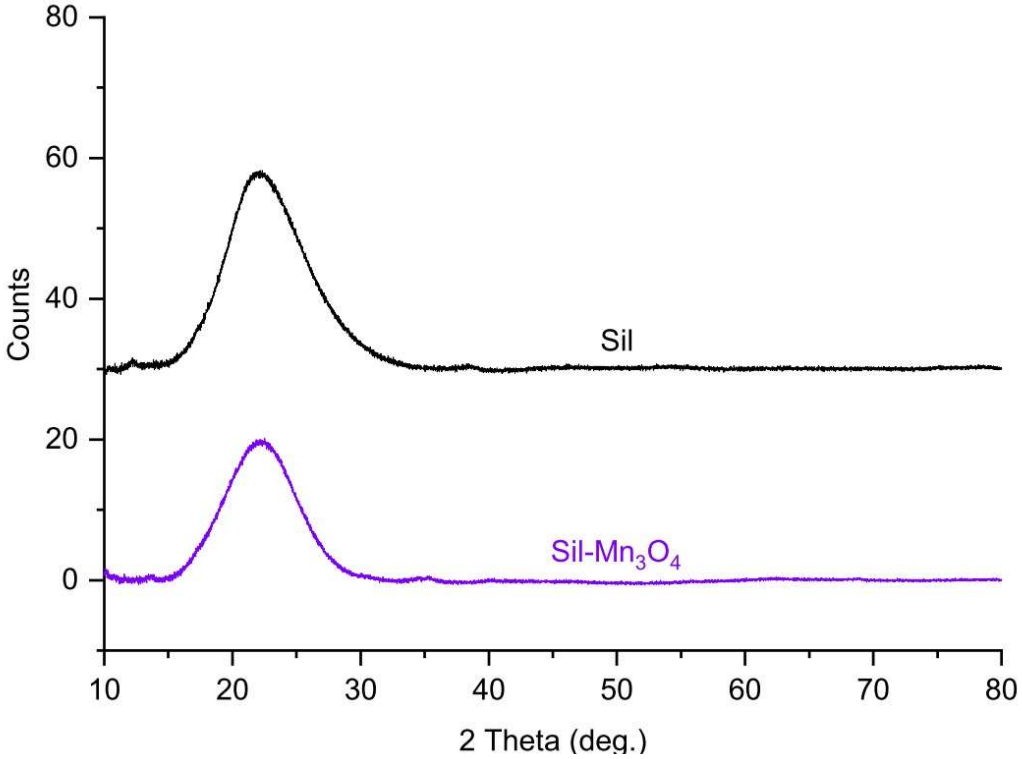

Figure S3. XRD patterns of spherical silica particles with Mn_3O_4 (SIL-Mn_3O_4), and reference material (spherical silica SIL).

References

[1] Laskowska, M.; Karczmarska, A.; Schabikowski, M.; Adamek, M.; Maximenko, A.; Pawlik, K.; Kowalska, O.; Olejniczak, Z.; Laskowski, Ł. Synthetic Opals or Versatile Nanotools—A One-Step Synthesis of Uniform Spherical Silica Particles. *Int. J. Mol. Sci.* **2023**, *24*, 13693.
